# Strengthening Community Antimicrobial Stewardship in Africa: A Systematic Review of the Roles, Challenges, and Opportunities of Community Health and Animal Health Workers

**DOI:** 10.12688/wellcomeopenres.24387.1

**Published:** 2025-07-09

**Authors:** Conrad Tumwine, Reuben Kiggundu, Fahad Lwaigale, Herman Mwanja, Hannigton Katumba, Mackline Hope, JP Waswa, Flavia Dhikusooka, Vivian Twemanye, Andrew Kambugu, Francis Kakooza, Dathan Byonanebye

**Affiliations:** 1 Centers, for Antimicrobial Optimization Network, Infectious Diseases Institute, Makerere University, Kampala, 256, Uganda; 2 Research, Makerere University Infectious Diseases Institute, Kampala, Central Region, 256, Uganda; 3 Global Health Security Department, Infectious Diseases Institute, Makerere University, Kampala, 256, Uganda; 4 Uganda National Institute of Public Health- Ministry of Health, Kampala, 256, Uganda; 5 Makerere University School of Public Health, Kampala, 256, Uganda

**Keywords:** Community Health Workers, Community Animal Health Workers, Antimicrobial Resistance, Antimicrobial Stewardship, One-Health, Africa

## Abstract

Antimicrobial resistance (AMR) remains a critical global health challenge, and is mainly due to inappropriate antimicrobial use in human and animal health sectors. This systematic review examines the roles of Community Animal Health Workers (CAHWs) and Community Health Workers (CHWs) in antimicrobial stewardship (AMS) across Africa where AMR burden is highest and AMS programs are limited.

Following PRISMA guidelines, this systematic review analyzed 16 studies (2017–2024) from nine African nations. We identified seven key roles of CAHWs and CHWs in AMS: 1) provision of clinical services (13 studies); 2) community mobilization (8 studies); 3) health promotion (7 studies); 4) provision of preventive services (5 studies); 5) epidemiological surveillance (4 studies); 6) advocacy (2 studies), and 7) medical waste management (2 studies). Despite their roles, challenges such as lack of supportive legislation (3 studies), inadequate remuneration (2 studies), and total reliance on foreign funding hinder AMS program sustainability. While most studies (14 studies) indicated that CAHWs and CHWs had received AMS training, their roles in the national AMR strategies remain unclear.

CAHW and CHWs could be leveraged in advancing health promotion, raising AMR awareness, supporting AMR surveillance, enhancing integrated management of diseases, and improving waste management within One Health frameworks. To realize this potential, there is a need to formalize CAHW/CHW roles through targeted legislation, specialized training and sustainable funding. This evidence highlights the critical need for policy reforms to harness their potential in strengthening health systems and curbing AMR across Africa.

PROSPERO registration number: CRD420251027215

## Plain Language

Antimicrobial resistance is a global health that is most pronounced in Africa, where the overuse and misuse of antibiotics among humans and animals is very common. This review set out to identify the roles of Community Health and Animal Health Workers in promoting responsible use of antibiotics (antimicrobial stewardship) across Africa. In this review that included 16 studies from nine African countries, published between 2017 and 2024, we noted that community health and animal health workers provided the following antimicrobial stewardship related services; clinical treatment, community mobilization, health promotion, preventative services, diseases surveillance, advocacy and medical waste management in the communities they served.

Despite the roles performed by community health and animal health workers, such programs face challenges such as poor funding frameworks, lack of enabling government policy nor legislation and poor provision of renumeration. Even though most articles reported training, their roles in antimicrobial resistance national action plans remain unclear.

These workers could play a bigger role in stopping the spread of AMR by educating people, helping track drug resistance, and supporting public health efforts. To do this, governments need to create clear laws, provide proper training, and secure local sustainable funding. The findings show that strong policies are needed to support these health workers in the fight against AMR in Africa.

## Introduction

Antimicrobial resistance (AMR) is a global one health challenge and is largely driven by inappropriate use of antimicrobials in both human and animal health ^1, 2^. The World Health Organization (WHO) Global Action Plan (GAP) and country specific National Action Plans (NAPS) for AMR containment provide guidance to implement antimicrobial stewardship (AMS) and combat AMR using a One Health approach ^3^. 

Community Animal Health Workers (CAHWs) are farmers or field agents selected by their communities, in collaboration with private veterinarians, public veterinary services, and supporting organizations such as projects and non-governmental organizations, to provide basic animal healthcare services and husbandry advice to livestock keepers ^4, 5^. According to a WHO Study Group, a community health worker (CHW) is a member of the community they serve, selected by that community, answerable to it, supported by the health system (but not necessarily part of its organization), and having shorter training than professional workers ^6, 7^. 

High prevalence of self-treatment and over-the-counter sales are key contributors of inappropriate antibiotic use in communities ^8, 9^. Drivers of self-treatment in agricultural communities are mainly structural deficiencies in the healthcare system such as limited access to healthcare facilities, medication stockouts and prolonged waiting times ^10^, all of which are currently being addressed by CAHWs and CHWs in most countries in Africa. The role of CAHWs and CHWs in implementing AMS interventions at the community level is crucial in reducing irrational antimicrobial use ^11^. As key public health points of contact, they promote health, support disease prevention, and dispense medicines when needed.

CAHWs and CHWs provide additional links between formal healthcare/veterinary care system and the community. Some generic modern-day tasks for CHWs and CAHWs have been synthesized and profiled into several categories: provision of preventive services ^12, 13^, provision of clinical services ^14– 16^, community mobilization ^17^, and epidemiologic surveillance, including record-keeping, and health promotion including health education ^18^. Furthermore, CAHWs and CHWs integrate One Health activities relating to zoonotic diseases, food safety and AMR ^19^ into national health systems across sub-Saharan Africa (SSA) outlining the complexity of AMR ^20, 21^.

The World Organization for Animal Health (WOAH) and WHO recommend CAHWs and CHWs as key elements in scaling up effective community-based health programs ^21, 22^. Indeed, several countries in Africa have relied on these community-based officers to increase access to health services. However, there is lack of guidance on the specific roles CHWs and CAHWs can play in the implementation of AMS programs at the community level ^23^. Therefore, the contributions of CAHWs and CHWs to AMR prevention remain largely unclear.

This systematic review aimed at identification CHWs and CAHWs roles in AMS according to the WHO GAP on AMR, as well as the identification of the facilitators, barriers for such AMS programs.

This systematic review identified the roles of CAHWs and CHWs in AMS for several key reasons. Firstly, it provides a comprehensive understanding of how these workers contribute to AMS initiatives. Secondly, it highlights the current gaps in existing AMS initiatives involving CAHWs and CHWs, informing future policy and practice. Thirdly, it facilitates the development of targeted training and support programs to enhance the effectiveness of CAHWs and CHWs in combating AMR at the community level. Ultimately, this systematic review serves as a foundational step towards integrating CAHWs and CHWs more effectively into national and global AMS strategies, ensuring a cohesive and impactful approach to addressing AMR. We conducted a systematic review to summarize the roles of CAHWs and CHWs in AMS in Africa.

## Methods

We conducted a systematic review of the existing literature on the role of CAHWs and CHWs in AMS. We limited the search to Africa because contextual factors are an important consideration influencing the character of stewardship interventions. The study protocol was informed by recommendations of Arksey and O’Malley ^24^ and the PRISMA Guidance for systematic reviews ^17^.

### Eligibility criteria

Studies were included if they described specific AMS interventions implemented by CAHWs or CHWs. Only articles with full-text availability and published in English between January 2017 and August 2024 were included in the review, as 2017 marks the period after the GAP was released by the WHO in 2016, prompted countries to develop their NAPs. Further, the inclusion criteria was based on the population, intervention, comparator, outcome (PICO) framework ^25^.

### Search strategy

We searched (a) electronic bibliographic databases; (b) grey literature, and (c) reference lists of included documents. Via Ovid, we searched MEDLINE, Global Health, CINAHL, Embase, Agricola, AGRIS and Web of Science databases. Search terms included: “Antimicrobial Resistance”, “Antimicrobial Stewardship”, “Community Health Workers”, “AMR Surveillance”, “antimicrobial surveillance”, “susceptibility testing”, “Role”, “CHWs”, “Community Animal Health Workers”, “CAHWs”, “antimicrobial use”, “Africa” and specific names of all African countries ( ***Appendix 1***). To preclude publication bias and to further explore the breadth of information on the topic, we conducted a grey literature search of Institutional Repositories of the World Health Organization (WHO), Food and Agriculture Organization of the United Nations (FAO), World Organization for Animal Health (WOAH), Africa Centre of Disease Control (CDC), country specific health ministries, and other renowned organizations. Finally, we screened reference lists of included documents for relevant articles.

### Data extraction, charting, synthesis, analysis, and presentation of results

After searching the primary sources, articles were exported from Endnote into Covidence for screening and analysis. A custom extraction tool was used during both the initial screening phase (to confirm study relevance) and the subsequent data extraction from the selected studies. To ensure systematic, reproducible study selection and data charting processes, and to foster high inter-rater reliability, a calibration exercise was undertaken.

A review team (RK, CT, HM, HK and FL) used a seminal article to ascertain if the extraction tool was appropriate. Once confidence in the tool had been established, authors (paired CT – HM and FL – HK) piloted and reviewed a sample of five papers, discussing any questions and further refining the extraction tool.

Articles meeting the inclusion criteria were then moved to the final stages of data extraction, charting, and synthesis. For data extraction and review, author pairs (CT – HK and FL – HM) independently extracted data in duplicate from each eligible article. All disagreements among the reviewers were resolved through discussion. The supervising reviewers (RK, HM) resolved any conflicts and provided oversight for the whole process.

Using the same approach, two independent reviewers (CT and HM) conducted a qualitative thematic analysis and any disagreements among the reviewers were resolved through discussion with the supervising reviewers (RK and HM). We appraised articles for quality using the CASP checklists ^26, 27^. Results were displayed in tables as descriptive statistics (frequencies and percentages).

## Results

**Figure 1.**
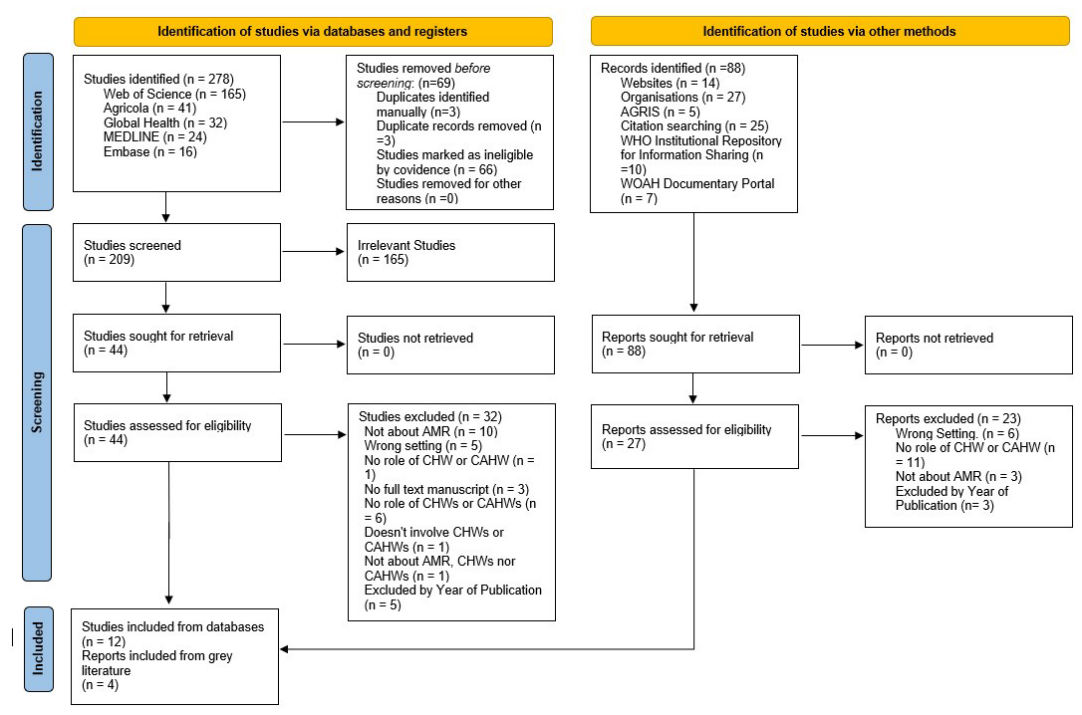
Systematic review PRISMA Diagram.

We identified 209 studies, of which 44 were deemed relevant for full-text screening ( Figure 1). Following assessment, 32 of these were excluded, leaving 12 studies in the final review. Additionally, out of the 88 records identified from grey literature, 27 full-text, non-peer reviewed health-related reports met the eligibility criteria for review, with 4 ultimately included in the final analysis. Reasons for exclusion are presented in Figure 1. In total, 16 articles were eligible for data extraction ( Table 1).

**Table 1.** Summary of studies included in the analysis.

Authors (and Publication year)	Title	Country of Study	Study Setting	Study design
**Community Health Workers.**				
Abuya T (2023) ^28^	Measuring implementation outcomes in the context of scaling up possible serious bacterial infection guidelines: Implications for measurement and programs	Kenya	Urban and Rural Communities	Cross-sectional
Iqbal (2021) ^29^	Embedding Community-Based Newborn Care in the Ethiopian health system: lessons from a 4-year programme evaluation	Ethiopia	Urban and Rural Communities	Quasi experimental
Wolde (2023) ^30^	Quality of sick child management by health extension workers: role of a complex improvement intervention	Ethiopia	Urban and Rural Communities	Quasi experimental
Ciccone (2024) ^11^	Point-of-care C-reactive protein measurement by community health workers safely reduces antimicrobial use among children with respiratory illness in rural Uganda: A stepped wedge cluster randomized trial	Uganda	Rural Communities	Randomized Clinical Trial
FAO, (2017) ^31^	Linking community-based animal health services with natural resource conflict mitigation in the Abyei Administrative Area	Abyei Administrative Area (AAA) *	Rural Communities	Cross-sectional
Musoke (2021) ^32^	Access, use and disposal of antimicrobials among humans and animals in Wakiso district, Uganda: a qualitative study	Uganda	Urban and Rural Communities	Cross-sectional
Gizachew, (2024) ^33^	Effect of community-based newborn care implementation strategies on access to and effective coverage of possible serious bacterial infection (PSBI) treatment for sick young infants during COVID-19 pandemic	Ethiopia	Rural Communities	Quasi experimental
Wakhumsama, (2022) ^34^	Togo Government Prioritizes Community Health as a National Development Plan	Togo	Rural Communities	Cross-sectional
**Community Animal Health Workers.**				
Bugeza (2017) ^35^	Participatory evaluation of delivery of animal health care services by community animal health workers in Karamoja region of Uganda	Uganda	Rural Communities	Cross-sectional
Fatmata Isatu Bangura (2022) ^36^	An Update on the Surveillance of Livestock Diseases and Antimicrobial Use in Sierra Leone in 2021—An Operational Research Study	Sierra Leone	Rural Communities	Cross-sectional
Konteh, (2023) ^37^	Improvement in the Surveillance System for Livestock Diseases and Antimicrobial Use Following Operational Research Studies in Sierra Leone January–March 2023	Sierra Leone	Urban and Rural Communities	Cross-sectional
Lemma (2019) ^38^	Community conversations on antimicrobial use and resistance	Ethiopia	Rural Communities	Cross-sectional
Mankhomwa (2022) ^39^	A Qualitative study of antibiotic Use Practices in Intensive Small-Scale Farming in Urban and Peri-Urban Blantyre, Malawi: Implications for Antimicrobial Resistance.	Malawi	Urban Communities	Cross-sectional
Chetail (2021) ^40^	Supporting Somalia’s Livestock Industry with Labelled Cash Transfer	Somalia	Urban and Rural Communities	Cross-sectional
**Community Health Workers and Community Animal Health Workers.**				
VSF International, (2023) ^41^	Why and how to integrate the One Health approach into training of Community Animal Health Workers	Somalia	Rural Communities	Unknown
Musoke (2020) ^42^	A One Health Approach to Strengthening Antimicrobial Stewardship in Wakiso District, Uganda	Uganda	Urban Communities	Cross-sectional

*Note: Abyei Administrative Area (AAA) is a currently contested zone located on the border between South Sudan and Sudan

### Study characteristics

Among the 16 articles reviewed, 12.5% (2/16) were published between 2017 and 2018, 31.3% (5/16) between 2019 and 2021, and the majority, 56.3% (9/16) were published between 2022 and 2024, indicating an increasing research interest in the topic over time. Ethiopia and Uganda had the highest number of studies with four from each of these countries. Other countries represented included; Sierra Leone (two studies), Kenya, Malawi, Somalia, Senegal and Somalia, Abyei Administrative Area (AAA), and Togo with one study each ( Table 2). Half of the studies were conducted in rural settings, 12.5% (2/16) in urban settings, and 37.5% (6/16) in both rural and urban communities. Of the 16 studies, 68.8% (11/16) employed cross-sectional designs, with 31.3% (5/16) using qualitative methods. Half of the studies focused primarily on CHWs, 37.5% (6/16) on CAHWs, and only 12.5% (2/16) on both CHWs and CAHWs ( Table 2).

**Table 2.** Characteristics of studies included in the analysis.

Characteristic	Frequency (n= 16)	Percentage (%)
**Year of Publication**		
2017 - 2018	2	12.5
2019 -2021	5	31.3
2022 - 2024	9	56.3
**Countries**		
Ethiopia	4	25.0
Uganda	4	25.0
Sierra Leone	2	12.5
Kenya	1	6.3
Malawi	1	6.3
Senegal and Somalia	1	6.3
Somalia	1	6.3
Abyei Administrative Area *	1	6.3
Togo	1	6.3
**Study Setting**		
Rural community	8	50.0
Urban and Rural Communities	6	37.5
Urban community	2	12.5
**Study Design**		
Cross Sectional	11	68.8
Quasi experimental	3	18.8
RCT	1	6.3
Unknown	1	6.3
**Study Population**		
CHWs	8	50.0
CAHWs	6	37.5
CHW and CAHWs	2	12.5

RCT: Randomized Clinical Trial, CHW: Community Health Worker, CAHW: Community Animal Health Worker, *Abyei Administrative Area (AAA) is a currently contested zone located on the border between South Sudan and Sudan

### Roles of community health workers and community animal health workers in antimicrobial stewardship in Africa

This review identified multiple roles that CHWs and CAHWs play in AMS). Seven key themes emerged from the thematic analysis: advocacy, community mobilization, epidemiological surveillance, medical waste management, provision of clinical services, provision of preventive services, and health promotion ( Table 3).

**Table 3.** Roles of CHWs and CAHWs in antimicrobial stewardship.

Role of CHWs and CAHWs in Antimicrobial Stewardship	n studies	Percentage % (n=16)	Citations
**Provision of clinical Services**	**13**	**81.3**	11, 28– 30, 32– 40, 42
Case Identification of sick animals or humans	8		11, 28– 30, 33, 34, 36, 37
Diagnosis of sick animals or humans	3		35, 38, 42
Dispensing of antibiotics	2		38, 39
Follow Up of sick animals or humans	3		28, 30, 34
Provision of surgical treatment to humans	1		35
Provision of treatment to animals or humans	9		28– 30, 32– 35, 40, 42
Referral of sick animals or humans	3		28, 30, 34
**Community Mobilization**	**8**	**50.0**	28, 29, 33– 35, 40– 42
Community mobilization	5		28, 29, 33, 35, 40
Infection prevention and control (IPC)	1		29
Safe use of chemicals and pesticides	1		41
Sustainable livestock production	1		35
Water, sanitation, and hygiene (WASH) promotion	2		34, 42
**Health Promotion**	**7**	**43.8**	28, 29, 33, 35, 39, 40, 42
Awareness about proper antibiotic use	1		42
Awareness of human health and animal health services.	3		29, 33, 40
Awareness on AMR, AMS and IPC	1		42
Health education about animal production	1		35
Health education about medicines	1		39
Health education on care for the sick	1		28
**Provision of preventative services**	**5**	**31.5**	29, 31, 34, 35, 40
Deworming of animals.	1		40
Health checkup of animals or humans	1		29
Prevention of external parasites	1		35
Vaccination of animals or humans.	4		31, 34, 35, 40
**Epidemiological Surveillance**	**4**	**25.0**	35– 37, 39
Passive AMR Surveillance	4		35– 37, 39
Report livestock statistics and movements	1		35
**Medical Waste Management**	**2**	**12.5**	32, 42
Medical Waste Management	2		32, 42
**Advocacy**	**2**	**12.5**	30, 41
Strengthening the linkages between health centres and the community health services.	2		30, 41

***Provision of clinical services.*** A significant proportion of the studies 81.3% (13/16) highlighted CAHWs and CHWs provision of clinical services as a key role. These included: 1) Provision of treatment ^28– 30, 32– 35, 40, 42^, as reported by a CHW in a study in Togo ^34^, that: *“I am also responsible for the …. management of life-threatening ailments in children under five and adults, management of certain childhood diseases such diarrhea, cough, and anemia … “* 2) Case identification ^11, 28– 30, 33, 34, 36, 37^ where one study noted CHWs’ use of new technologies in identification of febrile acute respiratory infection (ARI) reduced antibiotic use.3) Proper diagnosis of livestock diseases ^35, 38, 42^, as reported in a study in Uganda ^35^, who stated that: *“CAHWs could diagnose any 3 livestock diseases of their choice, based on clinical signs.”* 4) Follow-up care ^28, 30, 34^, as reported by a CHW in a study in Togo ^34^: *" I am also responsible for..... case finding and follow-up of tuberculosis patients in the community, …. "* 5) Dispensing medications ^38, 39^, CAHWs participation in dispensing medicines in communities reduced antibiotic misuse, 6) Surgical treatment support ^30^ where CAHWs performed minor surgeries such as wound dressing and castration. and 7) Referral services ^28– 30, 32– 35, 40, 42^.

***Community mobilization.*** Eight (8) studies reported community mobilization as a function of CAHWs and CHWs. Community mobilization was also done for infection prevention and control (IPC), safe use of chemicals and pesticides, sustainable livestock production and water, sanitation, and hygiene (WASH) promotion. Five studies specifically described their efforts in mobilizing communities for AMS initiatives ^28, 29, 33, 35, 40^. This is exemplified by a participant in the study in Kenya ^28^ who stated that; *“The endline assessment shows that most community health volunteers continued to provide services such as ... supporting providers in convincing those with severely sick young infants (SYI) to accept admission, and assisting caregivers in the identification of danger signs.”*

One study in Ethiopia ^29^ reported the role of CHWs in IPC efforts. In this study, CHWs educated communities on new born care and infection prevention to avert antibiotic use.

Similarly, CAHWs educated livestock farmers on the safe use of chemicals and pesticides, thereby minimizing harmful residues that could contribute to antimicrobial resistance ^41^, as reported by one study: *"AVSF facilitates …. CAHWs in risk perception and good management practices of chemicals, pesticides, and human and veterinary medicines."*

Community mobilization for sustainable livestock production was another key role noted among CAHWs, who help farmers adopt best practices for disease prevention ^30^, as reported by a participant in a study in Uganda ^35^; *"they [World Vision] normally encourage farmers to consult CAHWs on animal production and health services because they play a significant role in this respect”*

Two studies done in Togo and Uganda ^34, 42^ highlighted the involvement of CHWs in WASH promotion, which is essential in reducing the spread of infections.

***Health promotion.*** Health promotion was a dominant theme, reported in seven studies. CAHWs and CHWs facilitated education and awareness programs covering multiple areas including; 1) Proper antibiotic use ^42^, 2) Awareness of available health services ^29, 33, 40^, 3) Awareness of AMR, AMS and IPC ^42^, 4) Education on animal production ^35^ and medicine use ^39^.

CAHWs provided education about medicines as highlighted by a study in Uganda ^42^ which stated that: *“Farmers accessed information and medicines (including vitamins, vaccinations, and antibiotics) from a range of sources. These included lead farmers, veterinary shops, and community-based animal health workers.”*

CHWs also provided health education on care for the sick ^28^, as reported by a participant in the study by Abuya ^28^ who stated that: *“For the small children. . . we always tell them that small children should not be treated at home, and if she displays any signs whatsoever. . . the child should be rushed to the hospital at once. . . so that she can get treatment . . . and a government hospital is the best.”*

***Provision of preventive services.*** Five studies reported the preventive roles of CAHWs and CHWs, including deworming ^40^, health check-ups ^29^, prevention of external parasites ^35^, vaccination programs ^31, 34, 35, 40^ all which reduced diseases and improved productivity. One such article reported: “Given the importance of livestock for the livelihoods of both communities, a window of opportunity was identified for CAHWs to provide animal health services, specifically through a vaccination campaign.” ^31^

***Epidemiological surveillance.*** Four studies documented contributions to disease surveillance. Four studies ^35– 37, 39^ described involvement of CAHWs in passive surveillance, particularly in reporting animal illnesses observed. One study ^35^ highlighted their role in monitoring livestock movement and statistics, crucial for disease control. For example a study by Konteh ^37^ reported CAHWs provided information regarding animal illness observed during their visits to farmers to the livestock assistants at the chiefdom level or the District Livestock Officer (DLO)/District Veterinary Officer (DVO), depending on proximity; the livestock assistants/inspectors (LAs/LIs) gathered and sent the data to the DLO/DVO every week.”

***Advocacy.*** CAHWs and CHWs played a critical role in advocacy efforts aimed at promoting AMS ^30, 41^. This was through strengthening the medicine supply chains and fostering a collaborative work approach among local actors. This finding is exemplified by a study by Wolde ^30^ who stated that:

*" …strengthening the linkages between health centers and health posts.... Although medicines were not directly supplied by the program under this strategy, activities were also planned to strengthen medicine supply to the health posts through the existing government system."*

***Medical waste management.*** Two studies ^32, 42^ found that CHWs played a role in ensuring safe medical waste disposal, as reported by one participant in the study by Musoke ^32^ who reported that: *" Some of us were given safety boxes where we keep the spoilt medicines. When the box gets full, one takes the responsibility to return it to the government health centre for disposal. The medicines we dispose in the boxes include both personal ones and those we use for treatment of sick children in the community.”*

Further analysis of roles of CAHW and CHW in AMS can be accessed in Appendix 2

### Facilitators and barriers for AMS programs by CAHW and CHWs

***Existence of an enabling policy or legislation.*** Among the 16 included studies, 43.8% (7/16) reported the availability of legislation supporting CHWs or CAHWs. Specifically, 31.3% (5/16) highlighted supportive legislation for CHWs, 6.3% (1/16) for CAHWs, and 6.3% (1/16) for both CHW and CAHWs. In contrast, 18.8% (3/16) studies indicated the absence of legislation for CAHWs, while no studies explicitly reported a lack of legislation for CHWs or for both CAHWs and CHWs. The legislative status was unknown in 37.5% (6/16) studies, with 18.8% (3/16) for CHWs, 12.5% (2/16) for CAHWs, and 6.3% (1/16) both CAHWs and CHWs ( Table 4).

**Table 4.** Remuneration, training and legislation for CHWs and CAHWs among included articles and reports.

Factor	CHW n (%)	CAHW n (%)	CHW & CAHW n (%)	Overall n (%)
**Existence of an enabling policy or Legislation (n =16)**				
Yes	5 (31.3)	1 (6.3)	1 (6.3)	7 (43.8)
No		3 (18.8)		3 (18.8)
Unknown	3 (18.8)	2 (12.5)	1 (6.3)	6 (37.5)
**Provision of Renumeration to CHWs or CAHWs** **(n =16)**				
Yes	4 (25.0)			4 (25.0)
No		2 (12.5)		2 (12.5)
Unknown	4 (25.0)	4 (25.0)	2 (12.5)	10 (62.5)
**Training of CHWs and CAHWs (n = 16)**				
Yes	8 (50.0)	4 (25.0)	2 (12.5)	14 (87.5)
No		1 (6.3)		1 (6.3)
Unknown		1 (6.3)		1 (6.3)

***Provision of Renumeration.*** Remuneration was reported in 25.0% (4/16) of studies, indicating that CHWs received compensation for their work. No studies explicitly stated that CAHWs or both CAHWs and CHWs were remunerated. The absence of remuneration was reported in 2 (12.5%) studies involving CAHWs. A total of 62.5% (10/16) of studies did not specify remuneration details, with 25.0% (4/16) unknown for both CAHWs and CHWs separately and 12.5% (2/16) unknown for both CAHW and CHW collectively ( Table 4).

***Training (during project implementation).*** Training for CHW or CAHW was reported in 87.5% (14/16) of studies. Specifically, 50.0% (7/16) of studies reported training for CHWs, 25.0% (4/16) for CAHWs, and only 12.5% (2/16) for both CAHWs and CHWs. The absence of training was reported in only 6.3% (1/16) of studies for CAHW, and one study (6.3%, 1/16) did not report on CAHW training ( Table 4).

***Financing for CAHWs and CHW programs.*** Among the 16 studies analyzed, 75% (12/16) reported funding for CHWs, compared to 50% (8/16) for CAHW. Foreign governments were the primary funding source in 50% (8/16) of the studies. Multilateral organizations and Non-Governmental Organizations (NGOs) each accounted for 12.5% (2/16) of funding. Private organizations contributed 18.8% (3/16) of total funding and a research institution, contributed 6.3% (1/16) of total funding. Universities provided 12.5% (2/16) of total funding. All university-funded projects involved hybrid financing, combining sources like foreign governments and private organizations. ( Table 5).

**Table 5.** Distribution of CHW and CAHW financing among 16 studies.

Donor Agency	CHWs (n =12) *		CAHWs (n=8) *	
	Frequency	Percentage (%)	Frequency	Percentage (%)
**Foreign Governments (8 Studies)**	**4**	**33.3**	**5**	**62.5**
**Multilateral Organization (2 studies)**	**2**	**16.7**	**1**	**12.5**
**Non-Governmental Organization (2 studies)**	**1**	**8.3**	**1**	**12.5**
**Private Organizations (3 studies)**	**3**	**25.0**		
**Research Institutions (1 Study)**			**1**	**12.5**
**University (2 studies)**	**2**	**16.7**		

*2 studies reported both CAHW and CHW roles. *Hybrid funding was reported in 3 studies, 2 of which involved collaborations between universities and foreign governments either combined or in partnership with another funding category.*

## Discussion

This systematic review on roles, challenges, and opportunities of community health and animal health workers in Africa suggests that CAHWs and CHWs play key roles in antimicrobial stewardship in the community. CAHWs and CHWs played vital roles in advocating for better health services, community mobilization, epidemiological surveillance, medical waste management, health promotion (including health education), provision of preventative and clinical services. Nearly half of the studies reviewed reported CAHW and CHWs participating in provision of clinical services, mainly in case identification and direct treatment. Despite widespread training of CAHW and CHW reported, there is insufficient remuneration, financing, and legislation to support community AMS programs in Africa. 

The roles noted in the review are similar to those in non-AMR CAHW and CHW programs implemented across Africa ^43, 44^ and globally ^45, 46^ where their impact has greatly reduced disease burden and improved access to life saving medicines and vaccines ^47^. While the number of professional health workers has increased, gaps remain in delivering adequate animal and human health services in communities ^48^, necessitating the need for CHWs and CAHWs in the provision of empirical clinical services. These services, including case identification and treatment, are guided by structured algorithms such as Integrated Management of Childhood Illnesses (IMCI) with clear referral pathways with which CHWs and CAHWs are already familiar with ^32^.

Despite limited access to human ^32^ and animal ^49^ health services in African countries, some studies have noted negative perceptions among professional health workers regarding the participation of CHWs and CAHWs in AMS initiatives, citing the need to limit their roles, as they may increase inappropriate antibiotic use and thus increase the burden of AMR ^50^. However, our review does not align with those concerns, as the majority of included articles reported that training known to improve knowledge, skills, attitudes, and practices among participants ^51, 52^ supports the effective contribution of CHWs and CAHWs to AMS initiatives.

Similar to the first large-scale CHW program in Dian Xian, China ^53^, articles in this review reported CAHW and CHW involvement in animal surveillance (involves livestock and animal illness reporting) and vaccination initiatives ^54^, that contribute to reducing AMR. Their roles have since evolved to include deworming of animals, proper management of medical waste, and advocacy for improved community health services and access to medicines, all of which promote appropriate antibiotic use.

The roles of CHWs and CAHWs varied according to depth of community engagement. Some workers provided both health promotion and community mobilization, while others focused only on health promotion activities such as health education. Although the impact of AMS strategies was felt in the majority of the articles, those that reported community mobilization demonstrated more impact, such as improved acceptance of hospital admissions for sick infants, improved identification of danger signs ^28^, and better management practices for chemicals, pesticides, and human and veterinary medicines ^31^.

The review identified poor legislative support for community health programs. In the animal health sector, the legislative frameworks remain fragmented globally, with varying levels of acknowledgement of CAHW such as full national legislation backed by a Bill, recognition based by supporting policies/guidelines without full national recognition and training and/or use by government services on an informal basis ^55^. Nevertheless, countries such as Kenya have taken steps to formally enact the Community Health Workers Act, 2022 ^56^, which formally recognizes and provides renumeration for community health workers. However, this Act does not adopt a One-health approach and remains silent on the roles of CAHWs. Although renumeration for CHWs and CAHWs was reported as inadequate in our review, various models of renumeration have been deployed across Africa ^57^, including public, private, and cooperative systems with performance-based incentives ^58^.

Our review indicates that funding for community AMS programs in Africa primarily relies on external sources, specifically foreign governments, with no mention of funding from African countries. This illustrates the high dependence on foreign countries for AMS and AMR containment initiatives. The US President’s executive order of January 2025 to freeze and subsequently cut donor funding ^59^ threatens to undermine progress achieved so far. This review underscores the fragility of such funding frameworks and the need to diversify funding sources to sustain health programs. Nevertheless, self-funding in Africa may be a far reach due to multiple constraints including ongoing armed conflicts, widespread poverty ^60^, and competing high-priority expenditure items that strain health budgets ^61^. 

This systematic review faced several limitations. First, the available literature was limited in scope with many studies being small-scale, qualitative, or unpublished reports. Many articles from African francophone countries written in French were excluded and thus underrepresented in this review data. Finally, most interventions in the CHW and CAHW AMS space are poorly documented and published.

### Recommendations

Based on our findings, we make the following recommendations. First, due to the absence or weak legal and policy frameworks for CHWs and CAHWs, we recommend that governments enact and/or enforce legislation that clearly defines their responsibilities, establishes certification programs, and creates career pathways to improve their credibility and effectiveness. In the animal health sector, legal frameworks should be strengthened to ensure full national recognition of CAHWs, as most countries currently have fragmented policies offering varying levels of support.

Second, in light of inadequate funding and renumeration, we recommend adopting diverse hybrid funding models that reduce reliance on foreign aid such as public-private partnerships and integration of CHW and CAHW programs into the respective African government fiscal budgets.

Third, because most studies reported participation of CHW and CAHWs in community-based health service delivery, we recommend the development of clear guidelines on their acceptable roles to streamline roles and responsibilities. Additionally, all CAHW and CHWs should be trained and capacitated to provide community mobilization campaigns towards AMR mitigation strategies.

## Conclusion

Overall, CHWs and CAHWs play a critical role in community AMS in several African Countries. Their combined contributions in providing advocacy, community mobilization, epidemiological surveillance, medical waste management, provision of clinical services, provision of preventive services and health promotion services is essential to the one health approach. The strategic rural location of CHW and CAHWs presents a great opportunity to improve antimicrobial resistance awareness and good antimicrobial stewardship best practices in underserved communities. However, to unlock their full potential, addressing existing challenges such as limited legislative support, limited funding and renumeration though policy reforms and better renumeration is important.

## Statements and declarations

### Ethics approval

This study was approved by the Infectious Diseases Institute Research Ethics Committee (IDI-REC-2023-67). No participants’ identification information was used to disseminate or publish the study results.

This systematic review was registered with PROSPERO on 21 May 2025 as, The Role of Community Health Workers and Community Animal Health Workers in Community Antimicrobial Stewardship in Africa: A Systematic Review under registration number CRD420251027215 ^62^.

### Consent for publication

Not applicable

## Data Availability

All data supporting the findings of this study are openly available via the Open Science Framework under the Creative Commons Zero (CC0) license at the following DOI: 10.17605/OSF.IO/FBGSN ^63^. The repository includes the full dataset, supplementary files, Critical Appraisal Skills Programme (CASP), the data extraction tool used during the review process, and the completed PRISMA Checklist (Systematic Review of CHW and CAHW) Data are available under the terms of the Creative Commons Zero “No rights reserved” data waiver (CC0 1.0 Public domain dedication).
