# Supplementary material for: Strengthening Community Antimicrobial Stewardship in Africa: A Systematic Review of the Roles, Challenges, and Opportunities of Community Health and Animal Health Workers
Source: Wellcome Open Res. 2025 Jul 9;10:346. [Version 1] doi: 10.12688/wellcomeopenres.24387.1 (PMC12411836; doi:10.12688/wellcomeopenres.24387.1)
Supplement: Peer review history

# Reviewer report: Reviewer response for version 1

**DOI:** 10.21956/wellcomeopenres.26890.r127561

**Published:** 2025-09-04

**Authors:** Ifeanyi Elibe Mba

**Affiliations:** 1 University of Ibadan, Ibadan, Oyo, Nigeria

This systematic review assessed the potential roles of Community Animal Health Workers (CAHWs) and Community Health Workers (CHWs) in antimicrobial stewardship (AMS) across Africa, where antimicrobial resistance (AMR) is a major health threat and AMS programs are limited. Overall, the study found that CAHWs and CHWs could significantly contribute to promoting health and raising AMR awareness, supporting AMR surveillance, enhancing integrated disease management, and improving waste management within a One Health framework.

 Below are some of my thoughts, which the authors might consider taking to heart and integrating into their manuscript.

 Community and animal health workers are vital to strengthening AMS in Africa, which bears the greatest burden of infectious diseases but also faces significant challenges. From my perspective, effective interventions such as targeted, periodic training, multidisciplinary collaboration, and stronger regulatory frameworks will be crucial for enhancing their capacity and delivering greater health benefits to the populace. Importantly, tailoring AMS strategies to local contexts and engaging communities are also key to achieving sustainable progress. Also, while it is important to strengthen AMS, building a functional AMR surveillance and clinical research framework across Africa is critical to improving the effectiveness of any stewardship program and ultimately improving public health, which is the central aim of all health interventions. Without a clear understanding of where resistance is emerging and how it is spreading, efforts will remain generalized and inefficient. Furthermore, medical interventions and public health measures are direct or indirect outcomes of research. Therefore, there is a need to bring health research closer to the community level and shift from fragmented projects toward a robust, workable community framework. Such a framework, combined with effective stewardship, would address AMR challenges at multiple community levels and yield substantial health benefits to the populace

 While the paper was well executed, a small concern I have is that the knowledge of Community Animal Health Workers and Community Health Workers regarding AMS was not considered—you can’t give what you don’t have. Perhaps this could have added more context to the story.

 “This systematic review aimed at identification CHWs and CAHWs roles in AMS according to the WHO GAP on AMR, as well as the identification of the facilitators, barriers for such AMS programs” – something is wrong. Please rewrite for clarity.

 Last paragraph contains some redundant statement. Please rewrite for better clarity why simplifying the major aim/objective of the work.

Are the rationale for, and objectives of, the Systematic Review clearly stated?

Yes

Is the statistical analysis and its interpretation appropriate?

Yes

If this is a Living Systematic Review, is the ‘living’ method appropriate and is the search schedule clearly defined and justified? (‘Living Systematic Review’ or a variation of this term should be included in the title.)

Yes

Are sufficient details of the methods and analysis provided to allow replication by others?

Yes

Are the conclusions drawn adequately supported by the results presented in the review?

Yes

Reviewer Expertise:

NA

I confirm that I have read this submission and believe that I have an appropriate level of expertise to confirm that it is of an acceptable scientific standard, however I have significant reservations, as outlined above.

# Reviewer report: Reviewer response for version 1

**DOI:** 10.21956/wellcomeopenres.26890.r127560

**Published:** 2025-09-02

**Authors:** Alaa Abouelfetouh, Michael Shehat

**Affiliations:** 1 Microbiology and Immunology, Alexandria University Faculty of Pharmacy, Alamein International University Faculty of Pharmacy, Alexandria, Alexandria Governorate, Egypt

In the manuscript entitled:” Strengthening Community Antimicrobial Stewardship in Africa: A Systematic Review of the Roles, Challenges, and Opportunities of Community Health and Animal Health Workers”, the authors conducted a comprehensive literature search for all the studies that the reported roles of Community Animal Health Workers (CAHWs) and Community Health Workers (CHWs) in antimicrobial stewardship (AMS) across Africa in the period of 2017-2014. The manuscript is clear and follows logical order, the authors showed the roles, challenges, and opportunities of community health and animal health workers in Africa. Their results would clearly benefit the scientific community especially in the light of lack of such studies in developing countries. They have set logical exclusion and inclusion criteria for studies to be included. However, the manuscript needs some improvement before being published

 -The authors are advised to use keywords not already present in the title.

 -Under the introduction: can the authors provide 1-2 sentences to show figures explaining the extent of the problem of AMR in Africa? 

 -The last sentence of the introduction “ We conducted a systematic review to summarize the roles of CAHWs and CHWs in AMS in Africa.” is redundant. I understand the authors’ wish to state the aim at the end of the introduction but this was already stated at the start of the that last paragraph so please edit to avoid redundancy. 

 -For consistency, the authors can add percentages describing the number of studies from each country, same as they did with the year of publication and the setting where the study was conducted.

 -The authors are requested to merge Tables 1 and 2 since many of the data presented in Table 2 can be seen in Table 1.

 Table 3: shouldn’t the number of studies describing the role of CHWs and CAHWs be 14 instead of 13?

 -Table 3: the percentage of the 5 studies reporting on provision of preventive services should be 31.3.

 -Table 3: can the authors add the countries where these roles were identified in?

 -Under existence of an enabling policy or legislation: it is enough to describe the cases where there was a policy or legislation. The authors needn’t describe the negative or unknown cases since this can be seen in the table. Same for provision of renumeration. The authors can choose instead to include quotes as they did with the role of CHWs and CAHWs.

 -Under training (during project implementation): please correct the figure 50% (7/16) to 50% (8/16).

 -Under conclusions: Please replace antimicrobial stewardship with AMS.

 -If any of the reviewed articles described the economic impact of the roles of CHWs and CAHWs, it would be good to comment on that here.

 -Based on the smaller number of studies describing the roles of CAHWs relative to CHWs, would the authors recommend more research focusing on the roles and/or services provided by CAHWs or CHWs and CAHWs combined in a One health spirit?

 -The authors can elaborate more on the studies that showed negative perceptions among professional health workers regarding the participation of CHWs and CAHWs in AMS initiatives. These studies cited the need to limit their roles, as they may increase inappropriate antibiotic use and thus increase the burden of AMR. 

 -The author can compare in their discussion the roles and challenges they found in Africa with the roles and challenges of CHWs and CAHWs in other developing countries and again in developed countries to identify any gaps or differences between Africa and other countries.

 -The authors should include in the discussion section, any other reviews or meta analysis that have discussed roles of CHWs and CAHWs in Africa. Or they can state that this is the first review to address that if there hasn't been any other studies done before.  

 -The authors indicated an increasing research interest in the topic over time evident in the increase of the number of papers over time. It would be interesting to show in a graph, the progress of each role of CHWS and CAHs over time, or what role evolved over time.

Are the rationale for, and objectives of, the Systematic Review clearly stated?

Yes

Is the statistical analysis and its interpretation appropriate?

Not applicable

If this is a Living Systematic Review, is the ‘living’ method appropriate and is the search schedule clearly defined and justified? (‘Living Systematic Review’ or a variation of this term should be included in the title.)

Not applicable

Are sufficient details of the methods and analysis provided to allow replication by others?

Yes

Are the conclusions drawn adequately supported by the results presented in the review?

Yes

Reviewer Expertise:

Antimicrobial resistance and molecular epidemiology

We confirm that we have read this submission and believe that we have an appropriate level of expertise to confirm that it is of an acceptable scientific standard.
